# Integrated Pound−Drever−Hall laser stabilization system in silicon

**DOI:** 10.1038/s41467-017-01303-y

**Published:** 2017-10-31

**Authors:** Mohamad Hossein Idjadi, Firooz Aflatouni

**Affiliations:** Department of Electrical and Systems Engineering, University of Pennsylvania, Philadelphia, PA 19104 USA

## Abstract

Low noise stable lasers have far-reaching applications in spectroscopy, communication, metrology and basic science. The Pound−Drever−Hall laser stabilization technique is widely used to stabilize different types of lasers in these areas. Here we report the demonstration of an integrated Pound−Drever−Hall system that can stabilize a low-cost laser to realize a compact inexpensive light source, which can ultimately impact many fields of science and engineering. We present an integrated architecture utilizing an electronically reconfigurable Mach−Zehnder interferometer as the frequency reference to reduce the frequency noise of semiconductor lasers by more than 25 dB and the relative Allan deviation by more than 12 times at 200 μs averaging time. Compared to the bench-top implementations, the integrated Pound−Drever−Hall system has significantly lower power consumption, less sensitivity to the environmental fluctuations and occupies an area of only 2.38 mm^2^. The photonic and electronic devices are integrated on a standard 180 nm complementary metal-oxide semiconductor silicon-on-insulator process.

## Abstract (web summary)

Frequency-stable laser systems are important for many applications, like optical communications, that require high precision and low noise. Here, Idjadi and Aflatouni demonstrate that the widely used Pound-Drever-Hall technique can be fully integrated on-chip, miniaturizing the stabilization setup.

## Introduction

A compact laser source with low frequency fluctuations is an important component in many applications such as optical communication^1^, spectroscopy^2^, atomic clock^3,4^, astronomy^5^ and metrology^6^. A key part of a semiconductor laser, directly affecting its stability, is the frequency selective component such as the resonator or the cavity. In semiconductor lasers, the fluctuations in the resonator are mainly caused by thermal variations of the cavity length and index of refraction as well as the elasto-optic effect^7–9^. Laser stabilization using optical feedback^10,11^, electrical feedback^12^ and electrical feed-forward techniques^13,14^ have been demonstrated. However, the most widely used method in laser stabilization is the Pound−Drever−Hall (PDH) scheme^2–6,15–19^ where the frequency of the laser is measured using an optical frequency reference and the error signal in the electrical domain is amplified, filtered and fed back to the laser to suppress the laser frequency fluctuations. Bench-top PDH systems have been demonstrated where a high-quality factor cavity or resonator in a carefully controlled environment is used to stabilize the laser frequency^17,20,21^. Despite excellent stabilization performance, these bench-top systems are bulky, expensive, power hungry and highly sensitive to environmental fluctuations.

Standard complementary metal-oxide semiconductor (CMOS) silicon-on-insulator (SOI) processes offer high-optical confinement, high yield, scalability to mass production and co-integration with standard electronic devices and circuits and therefore are a suitable platform for monolithic integration of electronic-photonic systems in the infrared regime^22–24^.

Here, we report the demonstration of an integrated PDH frequency stabilization system based on an architecture where an electronically reconfigurable Mach−Zehnder interferometer (MZI) is used as the frequency reference. With a careful design of the on-chip optical delay line and the implemented electronically adjustable phase-control and loss-matching mechanisms in the MZI, a large extinction ratio and hence a high-frequency selectivity is achieved while the entire integrated PDH system only occupies an area of 2.38 mm^2^. In comparison with the bench-top implementations, where a high-quality factor resonator or cavity in a well-controlled environment is used, the small temperature gradient across the integrated PDH system significantly reduces the sensitivity to the environmental fluctuations which is a key advantage for an integrated PDH stabilization system. In addition, the use of a highly reconfigurable MZI instead of an integrated resonator greatly reduces the sensitivity to the process variations^25,26^ resulting in higher fabrication yield. Benefiting from the low noise design of the integrated electronic blocks and the high-extinction ratio of the integrated reconfigurable MZI, the frequency noise of a commercially available distributed feedback (DFB) laser is reduced by more than 25 dB while no temperature control for the integrated PDH system is used. The stabilized DFB laser exhibits more than 12 times relative Allan deviation reduction at 200 μs averaging time. All passive and active photonic components are designed and co-integrated with electronic devices on the GlobalFoundries GF7RFSOI process, a standard 180 nm CMOS SOI technology, with no post-processing.

## Results

### Integrated PDH principle of operation

**Fig. 1:**
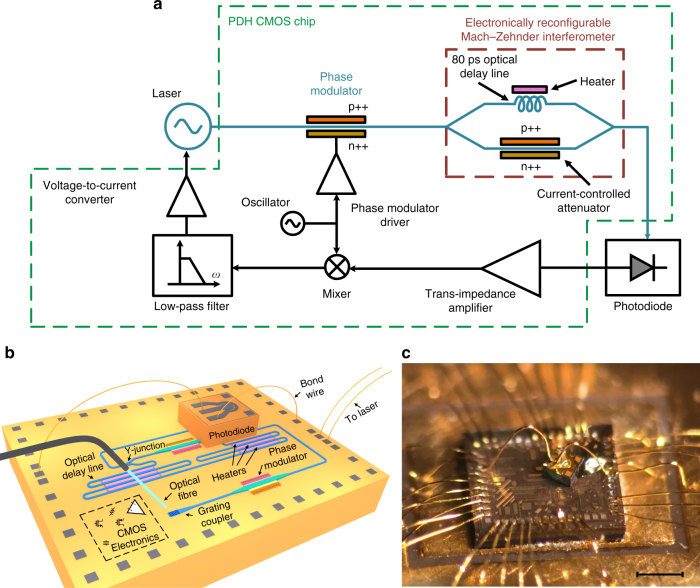
The integrated Pound−Drever−Hall laser frequency stabilization system. **a** The block diagram of the reported Pound−Drever−Hall (PDH) chip implemented in 180 nm GF7RFSOI CMOS silicon-on-insulator process. The laser output is phase modulated using a p-doped-intrinsic-n-doped (PIN) phase modulator, passed through the electronically reconfigurable Mach−Zehnder interferometer (MZI) with 80 ps delay imbalance, and photo-detected. The photo-current is amplified and converted to a voltage using a trans-impedance amplifier (TIA) and mixed with the same electrical signal used to phase modulate the laser. The mixer output is filtered, amplified, converted to a current and injected to the laser gain section to lock the laser to the notch in the MZI frequency response. The output of the voltage-to-current converter, the read-out signal, is asymmetric with respect to the frequency of the notch in the MZI frequency response, $f_{{\rm{notch}}}$. Therefore, the read-out signal indicates both the difference between the laser frequency and $f_{{\rm{notch}}}$ and whether the laser frequency is greater or less than $f_{{\rm{notch}}}$ (Supplementary Note 1). Note that the modulation sidebands are essential to detect the sign of the read-out (error) signal required to tune the laser toward $f_{{\rm{notch}}}$. **b** The structure of the reported PDH system. **c** Micro-photograph of the CMOS chip with a photodiode mounted on top. The details of the integrated CMOS chip are included in Supplementary Note 5 and Supplementary Fig. 6. The hybrid integration of the photodiode chip with the CMOS chip is discussed in Supplementary Note 8. Scale bar, 0.5 mm

Figure 1a shows the conceptual block diagram of the reported integrated PDH system. An electrical local oscillator (LO) is used to phase modulate the output of the laser using a p-doped-intrinsic-n-doped (PIN) modulator creating sidebands around the lasing frequency. The phase modulated signal passes through the electronically reconfigurable MZI acting as the frequency selective component. The MZI output is photo-detected and the photo-current is amplified and converted to a voltage using a trans-impedance amplifier (TIA) and mixed with the output of the LO. The mixer output is low-pass filtered, amplified, converted to a current using a voltage-to-current converter (VtoI) and injected to the gain section of the laser. As discussed in Supplementary Note 1, the VtoI output current, the read-out signal, is asymmetric with respect to the frequency of the notch in the MZI frequency response, $f_{{\rm{notch}}}$. Therefore, the read-out signal indicates both the difference between the laser frequency and $f_{{\rm{notch}}}$ and whether the laser frequency is greater or less than $f_{{\rm{notch}}}$.

Figure 1b shows the structure of the implemented PDH system. The laser is coupled into the CMOS chip using a grating coupler, and guided using nanophotonic waveguides to a PIN modulator. The modulated signal is guided to the reconfigurable MZI. The MZI output is backside-coupled to a vertical InGaAs photodiode (which is hybrid integrated with the CMOS chip) using a grating coupler. The output of the photodiode is wire-bonded to the input of the electronic circuits on the CMOS chip for further processing. The output of the chip is fed back to the laser to close the PDH loop. Figure 1c shows the micro-photograph of the implemented PDH system. All electronic and photonic devices are monolithically integrated on the GF7RFSOI CMOS SOI process except for the photodiode which is hybrid integrated (vertically) with the CMOS chip. The details of the integrated CMOS chip are included in Supplementary Note 5 and Supplementary Fig. 6. The hybrid integration of the photodiode chip with the CMOS chip is discussed in Supplementary Note 8.

### Design and characterization of the photonic components

**Fig. 2:**
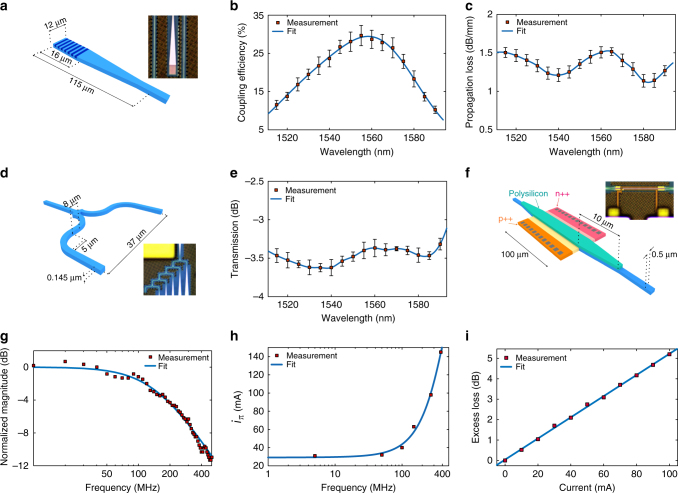
Characterization of photonic devices using on-chip test structures. **a** The test structure of the grating coupler. **b** The coupling efficiency of the grating coupler as a function of wavelength for coupling angle of 17°. **c** The measured propagation loss of a 1 mm long 500 nm wide waveguide after de-embedding the effect of grating couplers. **d** The structure of the implemented Y-junction. **e** Measured transmission of the implemented Y-junctions vs. wavelength. **f** The structure of the implemented p-doped-intrinsic-n-doped (PIN) phase modulator (Supplementary Fig. 2). **g** The frequency response of the PIN phase modulator where a 3-dB bandwidth of 150 MHz is estimated. **h** The modulator *i*_*π*_ vs. frequency. At each frequency point, the modulator *i*_*π*_ was measured by gradually increasing the modulator current and monitoring the modulator output optical power (Supplementary Note 2). An *i*_*π*_ of about 64 mA at 150 MHz is estimated. **i** The excess loss of the PIN modulator vs. electrical current injected into the PIN device. The unbiased PIN phase modulator has a measured insertion loss of 5.8 dB. Error bars represent the standard deviations of the measured data for 8 chips fabricated in two different GF7RFSOI runs

We have designed and characterized several active and passive photonic components required for implementation of the integrated PDH system on the GlobalFoundries GF7RFSOI process. Figure 2a shows the structure of the implemented grating coupler with measured 28% average peak efficiency at 1550 nm (Fig. 2b). The measured propagation loss of a 1 mm long 500 nm wide nanophotonic waveguide is depicted in Fig. 2c. The average excess loss of the implemented Y-junction in Fig. 2d is about 0.5 dB as depicted in Fig. 2e. Note that the error bars in Fig. 2b, c, e represents the standard deviations of the measured data for 8 chips fabricated in two GF7RFSOI runs. Figure 2f shows the structure of the implemented PIN phase modulator. The electro-optical frequency response of the PIN modulator is shown in Fig. 2g where a 3-dB bandwidth of about 150 MHz was measured. The current required to generate a $\pi$ radians optical phase shift across the PIN modulator, $i_\pi$, was measured at different frequencies, which is depicted in Fig. 2h. The modulator $i_\pi$ measurement details are included in Supplementary Note 2. The implemented PIN modulator can also be used as a current-controlled attenuator. Figure 2i shows the excess loss of the PIN modulator vs. input DC current where an average attenuation of 0.05 dB/mA was measured. More details on PIN modulator/attenuator measurements are included in Supplementary Note 2. Despite successful implementation of several active and passive photonic devices on the GF7RFSOI process, a wideband photodiode with high responsivity was not implemented as no material with efficient absorption coefficient in the 1550 nm range is available in this standard electronic process.

### The electronically reconfigurable MZI

**Fig. 3:**
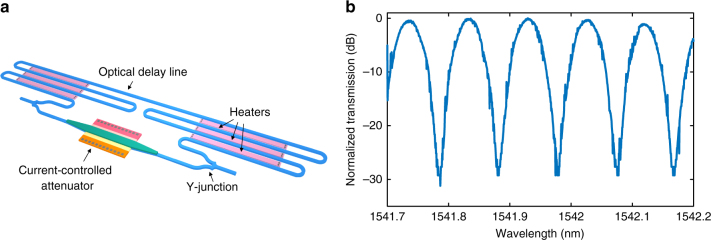
The implemented electronically reconfigurable Mach−Zehnder interferometer. **a** The structure of the implemented reconfigurable Mach−Zehnder interferometer (MZI). The delay difference between the two arms is about 80 ps. The loss mismatch can be compensated using the p-doped-intrinsic-n-doped structure acting as a current-controlled attenuator. The relative phase between the two arms can be adjusted using p-type polysilicon heaters placed between the waveguides of the delay line. **b** The measured transmission spectrum of the integrated MZI. The free spectral range of 0.1 nm and an extinction ratio of 28 dB were measured. The measurement details and the characterization setup are included in Supplementary Note 3 and Supplementary Fig. 4, respectively

Figure 3a shows the structure of the implemented electronically reconfigurable MZI serving as the frequency reference for the reported integrated PDH system. Two Y-junctions are used as the splitter and combiner at the input and output of the MZI, respectively. An optical delay line, which is implemented using 500 nm wide waveguides, is placed in the top arm of the MZI. The phase difference between the two arms of the MZI can be adjusted using p-type polysilicon heaters placed between the waveguides of the delay line. The phase difference between the two arms and the loss of the bottom arm can be adjusted independently to maximize the extinction ratio.

Figure 3b shows the measured response of the MZI. The measured free spectral range (FSR) of 0.1 nm corresponds to an 80 ps delay difference between the arms. By minimizing the loss mismatch between the two arms using the current-controlled attenuator and adjusting the relative phase between the two arms using the heaters, an extinction ratio of more than 28 dB was measured. The measurement details are included in Supplementary Note 3.

### The PDH open-loop read-out signal

**Fig. 4:**
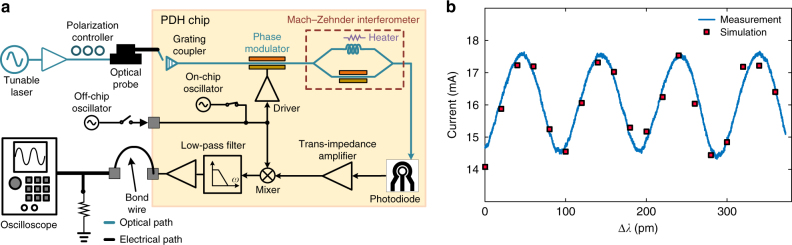
Open-loop measurement. **a** The block diagram of the Pound−Drever−Hall (PDH) open-loop measurement setup. An optical probe is used to couple the amplified output of an Agilent 81642A laser into the PDH chip. The output current of the voltage-to-current converter is used to drive a load emulating the impedance of the laser gain section and is monitored using an oscilloscope. **b** The simulated open-loop PDH read-out current (at $A_i$ = 82 dB) is shown which follows a sinusoidal form as predicted by eq. (1). The measured PDH open-loop read-out signal is also shown which is in close agreement with the simulation for the same operating point setting

To calculate the open-loop read-out signal at the output of the VtoI, the loop in Fig. 1a is disconnected after the VtoI. In this case, as discussed in Supplementary Note 1, the PDH read-out signal is calculated as

1 $$i_{{\rm{out}}} = K_{{\rm{OE}}} \times \sin \left( {\omega _0\tau + \phi } \right),$$

where $\omega _0,\tau$ and $\phi$ are the laser frequency, the delay difference between the arms of the reconfigurable MZI and the relative phase between the two arms, respectively. Also, the amplitude of the PDH read-out signal, $K_{{\rm{OE}}},$ depends on the optical and electrical settings and parameters and is defined in Supplementary Eq. (11). The electronic current gain, $A_1 = 20{{\log}}( {\frac{{i_{{\rm{out}}}}}{{i_{{\rm{PD}}}}}} )$, is defined as the ratio of the VtoI output current to the photodiode photo-current. This current gain can be adjusted between 52 and 112 dB. The measured performance of the photonic test structures were used to implement VerilogA models which together with the available scalable models of the electronic devices can be used for system simulations using Cadence tools^27^. Figure 4a shows the block diagram of the open-loop measurement setup. An optical probe is used to couple the amplified output of an Agilent 81642A tunable laser into the chip through a grating coupler. The output current of the VtoI is used to drive a load emulating the impedance of the laser gain section and is monitored using an oscilloscope. The measured read-out signal is compared with the simulated open-loop read-out signal in Fig. 4b. As predicted by eq. (1), when the wavelength of the tunable laser is swept, a sinusoidal waveform appears on the oscilloscope which is in agreement with the simulation for the same operating point.

### The PDH closed-loop operation and frequency noise reduction

Consider the case that the laser frequency is perturbed around its steady-state. When this small perturbation is applied to the proposed PDH system, it causes the PDH read-out signal to deviate from its steady-state. In this case, eq. (1) is modified to

2 $$i_{{\rm{out,ss}}} + \delta i_{{\rm{out}}} = K_{{\rm{OE}}} \times \sin [\left( {\omega _{{\rm{0,ss}}} + \delta \omega } \right)\tau + \phi ],$$

where $\omega _{0,{\rm{ss}}}$, $\delta \omega$, $i_{{\rm{out}},{\rm{ss}}}$ and $\delta i_{{\rm{out}}}$ are the steady-state laser frequency, the frequency perturbation, the steady-state output current and the output current change due to frequency perturbation, respectively. Since perturbation is by definition small, $\sin \left( {\delta \omega } \right) \approx \delta \omega$ and $\cos \left( {\delta \omega } \right) \approx 1$ can be considered. Therefore, eq. (2) is simplified to

3 $$\delta i_{{\rm{out}}} \approx K_{{\rm{OE}}}\cos \left( {\omega _{0,{\rm{ss}}}\tau + \phi } \right)\delta \omega \tau .$$

Using eq. (1) at steady-state, eq. (3) results in

4 $$\frac{{\delta i_{{\rm{out}}}}}{{\delta \omega }} \approx \tau K_{{\rm{OE}}},$$

where $\frac{{\delta i_{{\rm{out}}}}}{{\delta \omega }}$ is the PDH small signal frequency to output current conversion gain, and $i_{{\rm{out}},{\rm{ss}}} \ll K_{{\rm{OE}}}$ is assumed.

A semiconductor laser can be modelled as a current-controlled oscillator^28^. In this case, the instantaneous frequency of the laser is written as

5 $$\omega \left( t \right) = \omega _0 + K_{\rm{L}}i_{{\rm{ctrl}}}\left( t \right) + \omega _{\rm{n}}(t),$$

where $\omega _0$, $K_{\rm{L}}$, $i_{{\rm{ctrl}}}\left( t \right)$ and $\omega _{\rm{n}}\left( t \right)$ are the lasing frequency, the laser gain, the control current injected to the gain section of the laser and the laser intrinsic frequency noise, respectively. By perturbing $i_{{\rm{ctrl}}}\left( t \right)$ around its steady-state in eq. (5), the laser small signal current to frequency conversion gain is calculated as

6 $$\frac{{\delta \omega _0}}{{\delta i_{{\rm{ctrl}}}}} = K_{\rm{L}},$$

where $\delta i_{{\rm{ctrl}}}$ and $\delta \omega _0$ are the laser control current perturbation and the frequency change due to the current perturbation, respectively. Once the loop is closed, the VtoI output current, the read-out signal, is injected to the laser (as the laser control current).

**Fig. 5:**
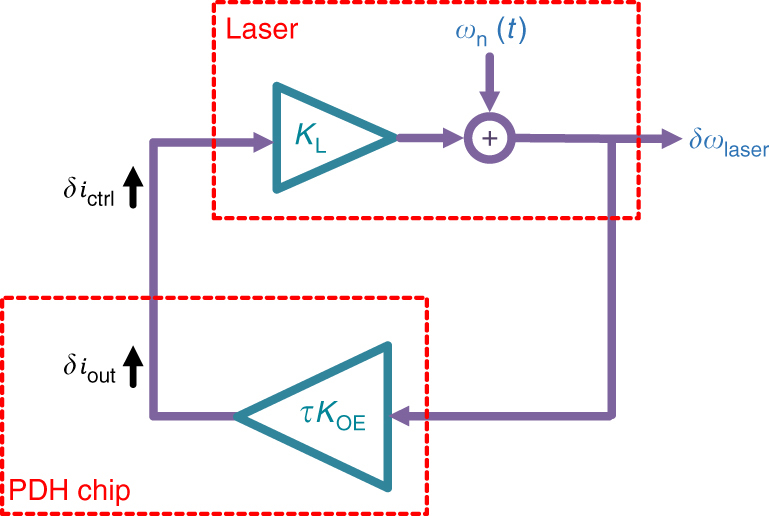
Linearized Pound−Drever−Hall system. The block diagram of the linearized Pound−Drever−Hall (PDH) system is shown where the laser and the PDH system are linearized around the operating point. When the loop is open, the total laser frequency noise, *δω*_laser_, is equal to the laser intrinsic frequency noise, *ω*_*n*_(*t*). When the loop is closed, the PDH chip output current, *δi*_out_, would be the same as the laser control current, *δi*_ctrl_. In this case, the laser frequency noise is reduced by the loop gain, where the loop gain is defined as the product of the PDH small signal frequency to output current conversion gain, *τK*_0E_, and the laser small signal current to frequency conversion gain, *K*_L_

Figure 5 shows the simplified linearized block diagram of the reported integrated PDH system. When the loop is disconnected after the VtoI, the total frequency noise at the laser output, $\delta \omega _{{\rm{laser}}}$, is equal to $\omega _{\rm{n}}(t).$ When the loop is closed, the total laser frequency noise is reduced by the loop gain as

7 $$\delta \omega _{{\rm{laser}}} = \frac{{\omega _{\rm{n}}(t)}}{{1 + K_{{\rm{Loop}}}}},$$

where the loop gain is defined as $K_{{\rm{Loop}}} = |\tau K_{{\rm{OE}}}K_{\rm{L}}|.$

Note that according to Supplementary Eq. (11), $K_{{\rm{OE}}}$ and hence the loop gain depends on the electrical phase shift, $\theta$, which can be adjusted electronically in the modulator driver circuit. Also, the gain of all electronic blocks can be set independently to adjust the loop gain.

**Fig. 6:**
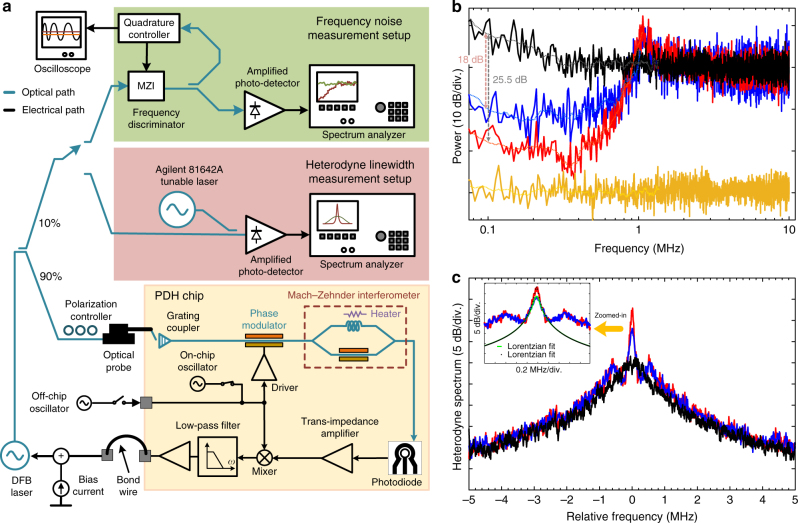
Frequency noise spectra and beat note spectral measurement. **a** The frequency noise and heterodyne linewidth measurement setups. For the frequency noise measurements, an optical fiber based Mach−Zehnder interferometer (MZI) is utilized as a frequency discriminator (Supplementary Note 4). The phase difference between the two arms of the MZI is set to 90^o^ using the quadrature controller. To measure the heterodyne linewidth of the distributed feedback (DFB) laser before and after Pound−Drever−Hall (PDH) stabilization, the beat note between the DFB laser and an Agilent 81642A tunable laser is monitored on an electrical spectrum analyzer. **b** Frequency noise measurement. The measured closed-loop response of the reported PDH system is shown where the original frequency noise of the laser (black) is reduced by more than 25 dB (for *A*_*i*_ = 112 dB) when an off-chip 150 MHz oscillator is used (red) and by more than 18 dB when the on-chip 280 MHz oscillator is used (blue). The noise floor of the measurement setup is shown in yellow. **c** The full-width at half-maximum (FWHM) linewidth measurement. The beat note between the DFB laser and an Agilent 81642A tunable laser is monitored on an Agilent 8563E electrical spectrum analyzer. Under the closed-loop condition, the linewidth of the free-running laser (black) is reduced using on-chip oscillator (blue) and off-chip oscillator (red). The inset shows the zoomed-in heterodyne spectra with Lorentzian fits

Using open-loop simulations for $A_{\rm{i}}$ = 112 dB, $K_{{\rm{Loop}}} \approx$ 18.8 was estimated which corresponds to a 25.9 dB frequency noise suppression. Figure 6a shows the experimental setup that was used for frequency noise and heterodyne linewidth measurements. The output of an Alcatel 3CN00325CW distributed feedback (DFB) laser is split into two branches using a 90/10 fusion coupler. In the bottom branch, 90% of the laser output power is coupled into the reported PDH chip after polarization adjustment. In the top branch, 10% of the laser power is used for frequency noise and linewidth monitoring. For the frequency noise measurements, an optical fiber based MZI acting as a frequency discriminator is used (see Supplementary Note 4 for more details). Figure 6a also shows the heterodyne linewidth measurement setup where the beat note between the DFB laser and an Agilent 81642A tunable laser is monitored on an Agilent 8563E spectrum analyzer. The full-width at half-maximum (FWHM) linewidth of 25 kHz is measured for the Agilent 81642A tunable laser which is used to estimate the linewidth of the DFB laser in the heterodyne setup.

Figure 6b shows the measured frequency noise of the DFB laser before and after PDH stabilization. Under the closed-loop condition, the original frequency noise of the DFB laser is reduced by more than 25 dB when the off-chip oscillator (at 150 MHz) is used and by more than 18 dB when the on-chip oscillator (at 280 MHz) is used. Note that while the frequency of the off-chip oscillator can be set to 150 MHz, the frequency of the on-chip oscillator cannot be set below 280 MHz as the oscillator parasitic capacitors were overestimated during the on-chip oscillator design. Based on Fig. 2g, changing the LO frequency from 150 to 280 MHz results in over two times loop gain reduction which is approximately consistent with 7 dB lower frequency noise suppression. The frequency of the on-chip oscillator can be lowered to 150 MHz by increasing the capacitance of the on-chip oscillator at no power consumption penalty.

The measured heterodyne spectrum of the DFB laser before and after frequency noise reduction is shown in Fig. 6c where the free-running FWHM linewidth of the DFB laser emitting 12 dBm at 194.68 THz was reduced from 750 kHz to 55 kHz when the off-chip oscillator was used (red) and to 95 kHz when the on-chip oscillator was used (blue). The resolution bandwidth for these measurements was set to 10 kHz. The zoomed-in heterodyne spectra with Lorentzian fits are illustrated in the inset of Fig. 6c.

We have calculated the approximate linewidth reduction of the DFB laser using the measured frequency noise spectra^29^ for free-running and closed-loop cases in Fig. 6b. The calculated linewidth reduction for the on-chip and off-chip oscillator cases are 11.9 dB and 9.8 dB, respectively, which is in agreement with the measurement results shown in Fig. 6c.

**Fig. 7:**
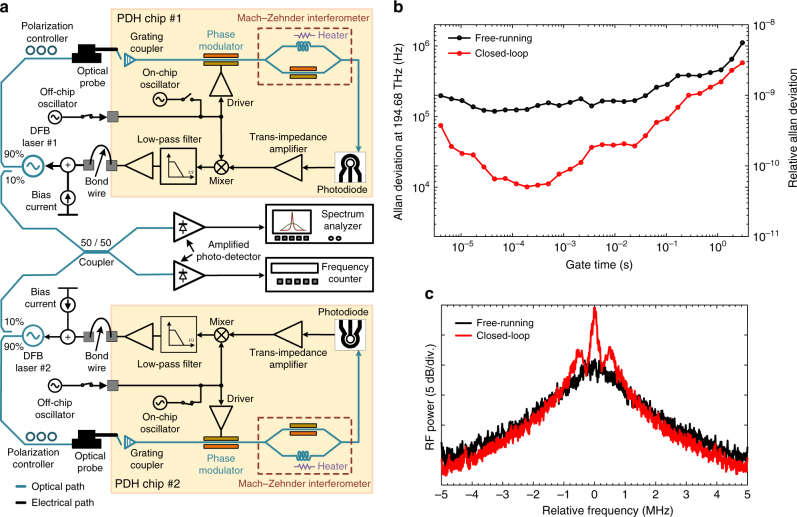
Allan deviation measurement. **a** The Allan deviation measurement setup. Two Alcatel 3CN00325CW distributed feedback (DFB) lasers were independently Pound−Drever−Hall (PDH) stabilized using two PDH chips. The outputs of two stabilized lasers were combined using a 50/50 coupler and photo-detected. The photo-current was amplified and used to measure the relative Allan deviation and the spectrum of the beat note. **b** The measured relative Allan deviation of the beat note between two DFB lasers is shown for free-running and independently PDH stabilized cases. At a gate time of 200 μs, the relative Allan deviation of the beat note is decreased from 6.5 $\times$ 10^−10^ to 5.2 $\times$ 10^−11^ when the PDH stabilization was engaged. In this case, assuming the two DFB lasers are equivalent, a relative Allan deviation reduction from 4.6 $\times$ 10^−10^ to 3.6 $\times$ 10^−11^ is estimated for each PDH stabilized laser. **c** The radio frequency (RF) spectrum of the beat note between two DFB lasers for free-running and closed-loop cases

To measure the frequency stability of the PDH stabilized laser, two CMOS chips were used to perform Allan deviation measurements in the setup depicted in Fig. 7a. Two Alcatel 3CN00325CW DFB lasers emitting 12 dBm at 194.68 THz were independently PDH stabilized using two CMOS chips. The outputs of two stabilized lasers were combined using a 50/50 coupler. Two output branches of the 50/50 coupler were photo-detected separately. The top branch was used to monitor the radio frequency (RF) power spectrum of the beat note between two DFB lasers and the bottom branch was used to measure the Allan deviation using a Tektronix FCA3120 RF frequency counter. Figure 7b shows the relative Allan deviation of the beat note measured over the range of gate times from 4 μs to 3 s before and after engaging the integrated PDH systems. Relative Allan deviation smaller than 10^−10^ was measured from 40 μs to 1 ms which is more than 10 times lower than that of the free-running case. Assuming two DFB lasers are independent and equivalent, the relative Allan deviation for each stabilized DFB laser over this range of gate times is lower than 5.5 $\times$ 10^−11^ (with a minimum of 3.6 $\times$ 10^−11^ occurring at 200 μs, corresponding to more than 12 times Allan deviation reduction). Figure 7c shows the RF spectrum of the beat note between two DFB lasers for both free-running and closed-loop cases where linewidth reduction is apparent. The resolution bandwidth for these measurements was set to 10 kHz resulting in a 50 ms sweep time for a frequency span of 10 MHz. Under the closed-loop condition, we have estimated the FWHM linewidth of 100 kHz for the beat note which, assuming equivalent and independent DFB lasers, corresponds to a FWHM linewidth of 50 kHz for each stabilized DFB laser which is in close agreement with the heterodyne linewidth measurements shown in Fig. 6c.

## Discussion

Ideally, the integrated PDH system should be able to perform a large frequency noise reduction over a wide bandwidth. However, undesired effects such as noise of electronic and photonic devices and the laser frequency modulation (FM) response limit the PDH stabilization performance. As discussed in Supplementary Note 7, while the system noise including the noise of electronic devices, the relative intensity noise of the laser and the photodiode shot noise are suppressed by the loop gain, the residual system noise is injected to the laser increasing its frequency noise. Therefore, reducing the system noise would improve the PDH stabilization performance. Another undesired effect is the FM response of the semiconductor laser which limits the frequency noise reduction bandwidth. The thermal and the charge carrier effects modulate the refractive index of the gain medium in opposite directions. As a result, an abrupt phase change occurs in the laser FM response^30^ (at about 0.9 MHz for the Alcatel 3CN00325CW DFB laser) that can cause loop instability for a large enough loop gain. This effectively limits the loop gain-bandwidth product. A lead-lag filter may be added to the PDH loop to improve the system phase margin allowing for a higher frequency noise reduction bandwidth at the same (or higher) loop gain.

In conclusion, we have demonstrated an integrated PDH system implemented on a 180 nm standard CMOS process without post-processing. In the proposed architecture, an electronically reconfigurable MZI is introduced serving as the frequency reference making the reported PDH system less sensitive to fabrication process variations. The implemented PDH system was used to suppress the frequency noise of a commercially available semiconductor lasers by more than 25 dB, its FWHM linewidth by more than 13 times and its relative Allan deviation by more than 12 times (at a 200 μs averaging time) while occupying a 2.38 mm^2^ area. The integrated PDH system can be used to significantly improve the noise performance and stability of a low-cost laser chip enabling realization of inexpensive highly stable compact light sources with many applications in science and engineering such as communication, sensing and metrology, to name a few.

## Methods

### CMOS chip implementation

The detailed block diagram of the CMOS chip is shown in Supplementary Fig. 6. All electronic and photonic devices except for the vertical InGaAs photodiode are monolithically integrated on the GlobalFoundries GF7RFSOI, a standard 180 nm CMOS SOI process, with no post-processing. The implemented photonic devices include the input grating coupler, a PIN phase modulator, nanophotonic waveguides and the reconfigurable MZI. The MZI output is photo-detected using a photodiode chip placed on top of the grating coupler. The photo-current is amplified and converted to a voltage using a multi-stage TIA. The TIA output is mixed with the LO signal using a double-balanced mixer. The LO signal is generated on-chip by a voltage-controlled ring oscillator and can be overwritten by an off-chip oscillator. The mixer output is amplified and low-pass filtered and converted to a current using a voltage-to-current converter. The CMOS chip details are presented in Supplementary Note 5 and the schematics of core electronic blocks are shown in Supplementary Fig. 7. The hybrid integration of the photodiode chip with the CMOS chip is discussed in Supplementary Note 8.

### Characterization of electronic blocks and the noise effect

The measured performance of the CMOS electronic blocks is summarized in Supplementary Note 6 and Supplementary Table 1. The noise sources and their effects are discussed in Supplementary Note 7. A list of all measurement equipment and components are provided in Supplementary Table 2.

### Data availability

The data that support the findings of this study are available from the corresponding authors upon reasonable request.

## Electronic supplementary material

Supplementary information
