# Supplementary material for: Integrated Pound−Drever−Hall laser stabilization system in silicon
Source: Nat Commun. 2017 Oct 31;8:1209. doi: 10.1038/s41467-017-01303-y (PMC5663923; doi:10.1038/s41467-017-01303-y)
Supplement: Supplementary file 1 — Supplementary information [file 41467_2017_1303_MOESM1_ESM.pdf]

Supplementary Figures

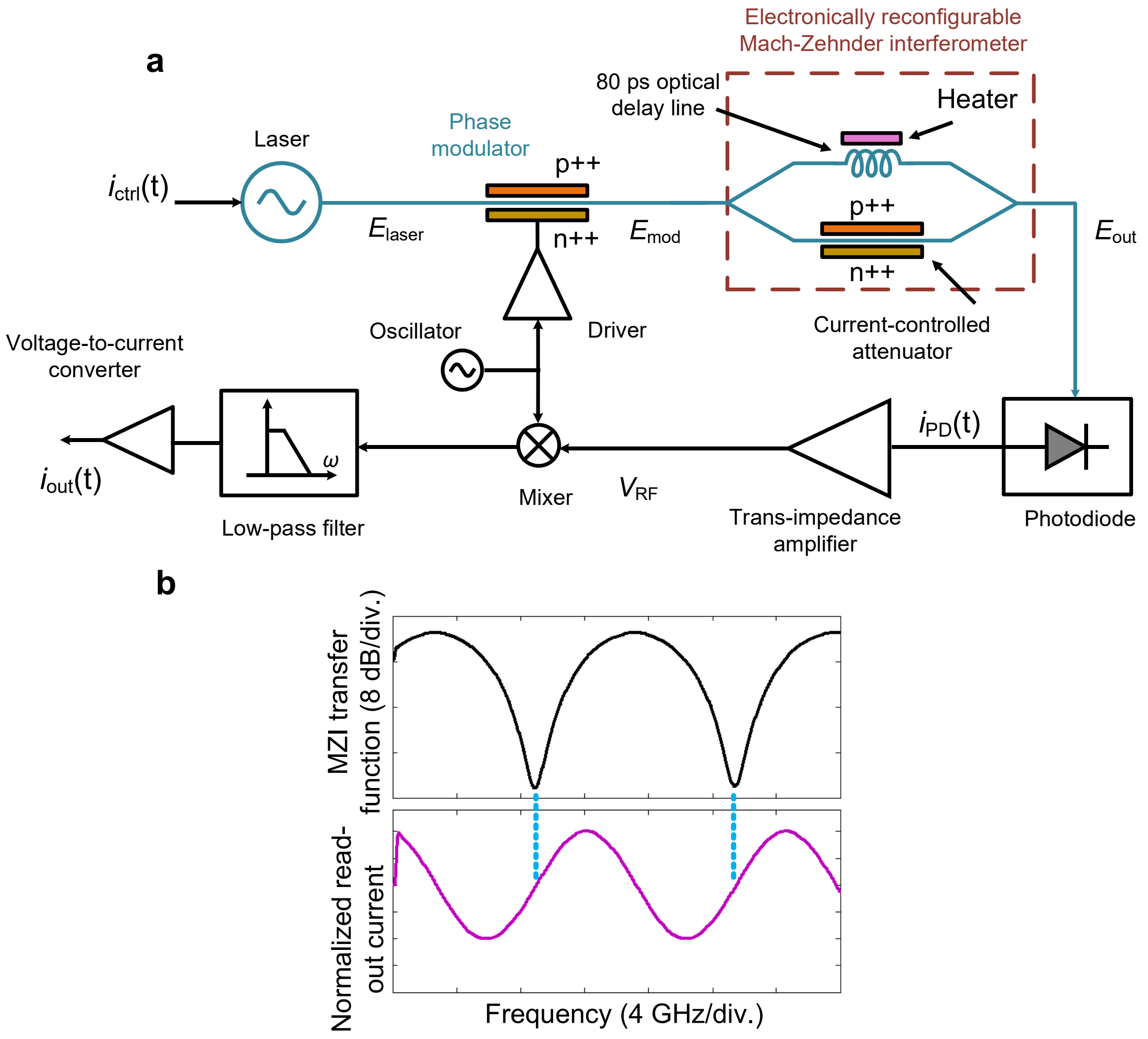

Supplementary Figure 1 | The open-loop Pound-Drever-Hall system and the read-out signal. (**a**) The block diagram of the pound-drever-hall (PDH) system under the open-loop condition. A small signal current, i_{ctrl}(t), is applied to the laser, changing the phase and frequency of the laser electric field. The laser output electric field, E_{laser}, is phase modulated and the modulated signal, E_{mod}, passes through the electronically reconfigurable Mach-Zehnder interferometer (MZI). The MZI output, E_{out}, is photo-detected. The photo-current, i_{PD}(t), is amplified and converted to a voltage using a trans-impedance amplifier (TIA). The TIA output, V_{RF}, is mixed with the oscillator output (which is also used to phase modulate the laser output electric field) and low-pass filtered. The filter output voltage is converted to the PDH read-out current, i_{out}(t), using a voltage-to-current converter. (**b**) The simulated magnitude response of the MZI transfer function (black) and the simulated normalized read-out current (purple). The read-out current is asymmetric around the notch frequency in the MZI transfer function.

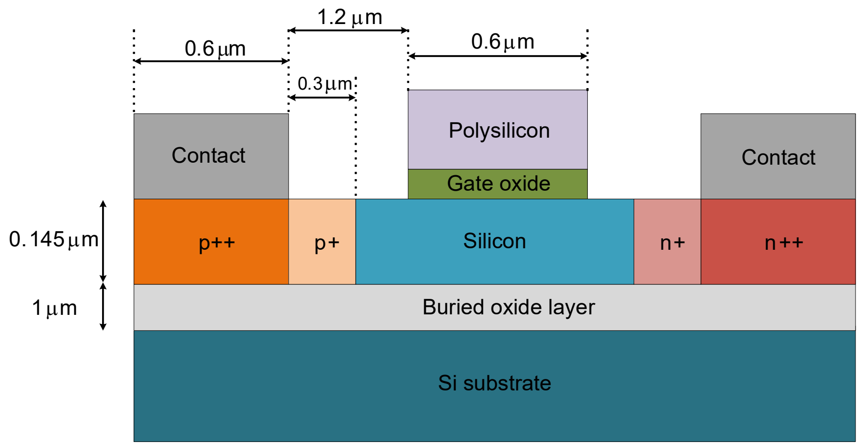

Supplementary Figure 2 | Cross-section of the implemented phase modulator. The optical mode is confined in the polysilicon and silicon (Si) regions. Moderately doped silicon regions (p+ and n+) are used to form the junction and heavily doped regions (p++ and n++) are used under the electrical contacts. The refractive index of the silicon waveguide (between p+ and n+ regions) changes through carrier injection modulating the phase of the optical wave.

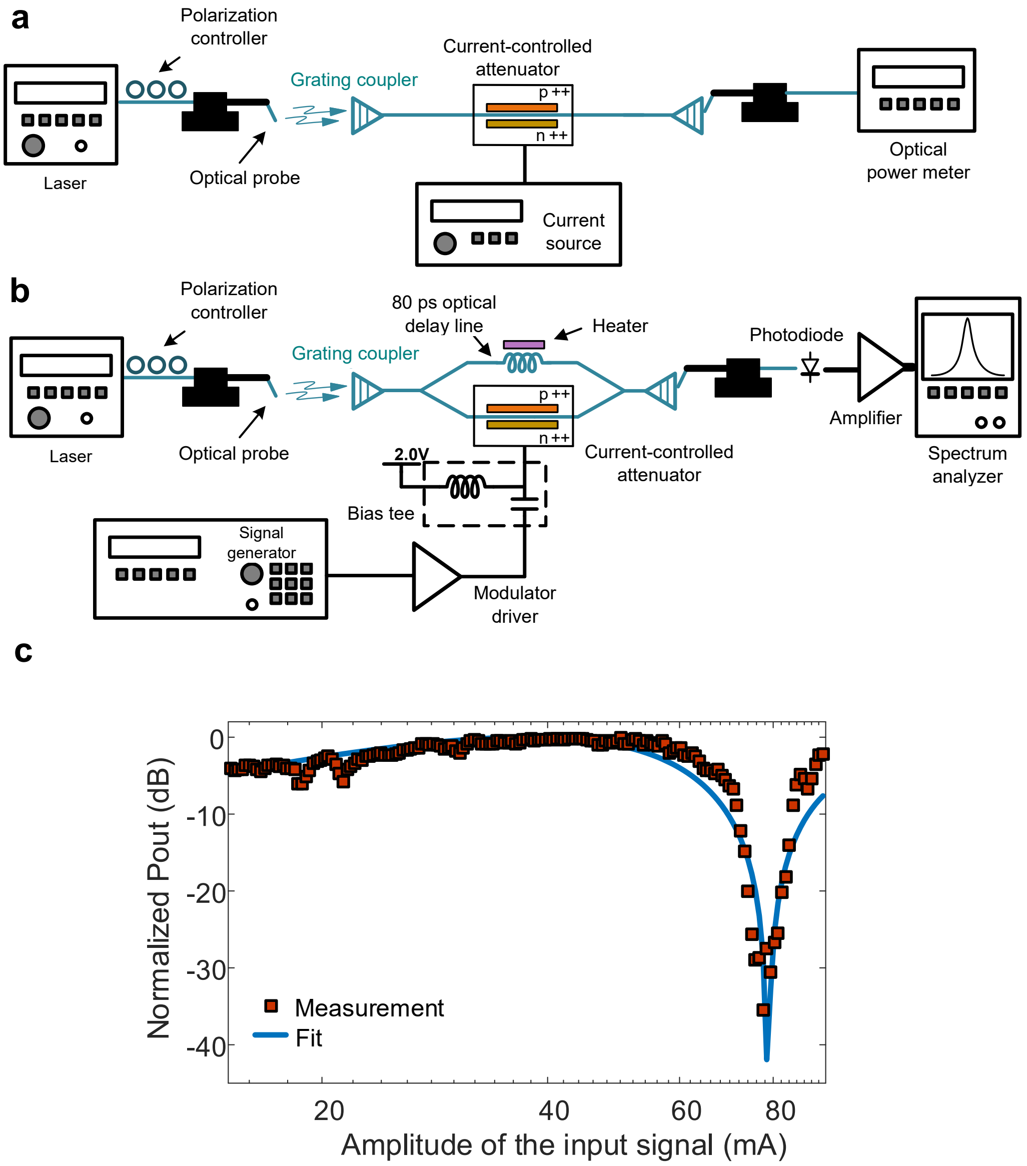

Supplementary Figure 3 | Phase modulator characterization. (**a**) The setup for characterization of the p-doped-intrinsic-n-doped (PIN) structure as a current-controlled attenuator. (**b**) The bandwidth and i_π measurement setup. (**c**) The modulator response versus amplitude of the input signal (at 150 MHz). By fitting a Bessel function of the first kind to the measured data, an i_π of 64 mA is estimated.

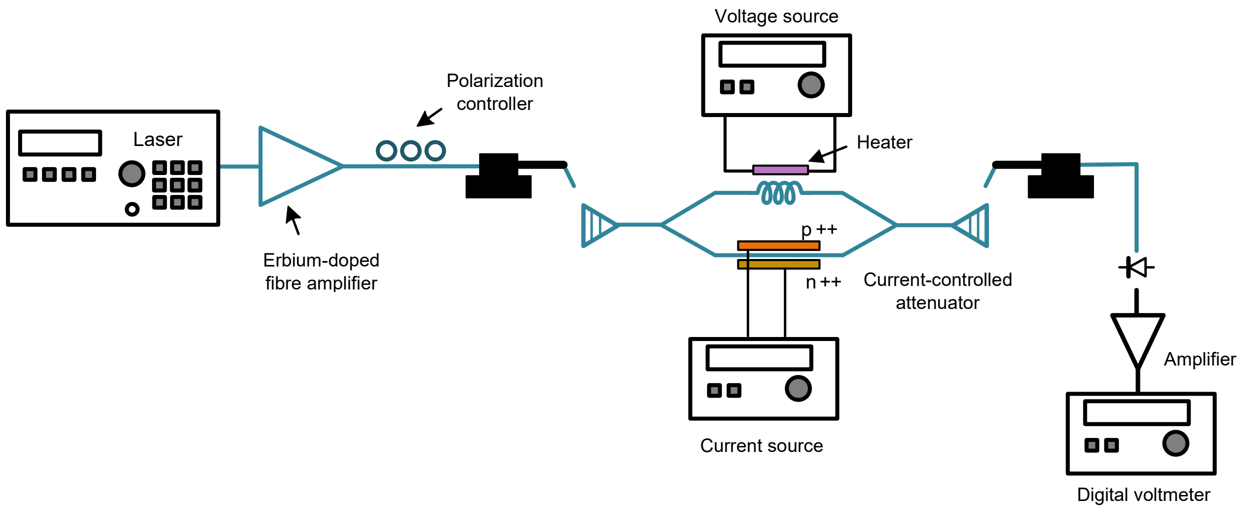

Supplementary Figure 4 | Measurement setup for electronically reconfigurable Mach-Zehnder interferometer characterization. The tunable laser output is amplified using an Erbium-doped fibre amplifier (EDFA) and is injected to the electronically reconfigurable Mach-Zehnder interferometer (MZI) after polarization adjustment. The MZI output is photo-detected, amplified and monitored on a digital voltmeter. To maximize the extinction ratio, the current injected to the current-controlled attenuator is adjusted minimizing the loss mismatch between the MZI arms. By setting the voltage applied to the heater, the relative phase between the MZI arms is adjusted.

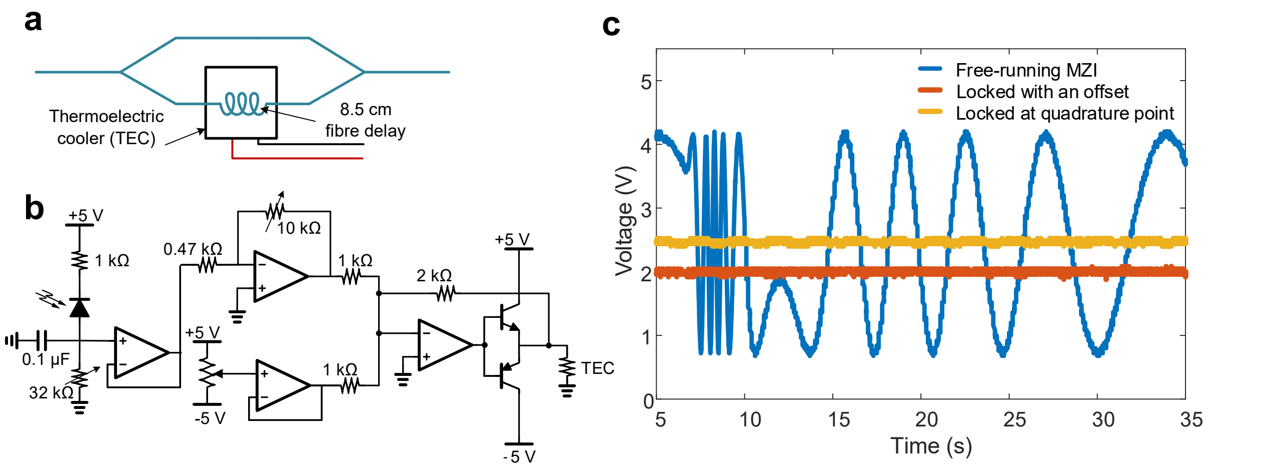

Supplementary Figure 5 | Frequency discriminator. (**a**) The schematic of the optical fibre based Mach-Zehnder interferometer (MZI) used as a frequency discriminator. The thermoelectric cooler (TEC) is used to thermally adjust the relative phase between the arms of the MZI. (**b**) The schematic of the electronic control unit used for relative phase adjustment between the arms of the off-chip frequency discriminator. (**c**) The voltage at the output of the control circuit corresponding to the relative phase between the arms of the frequency discriminator.

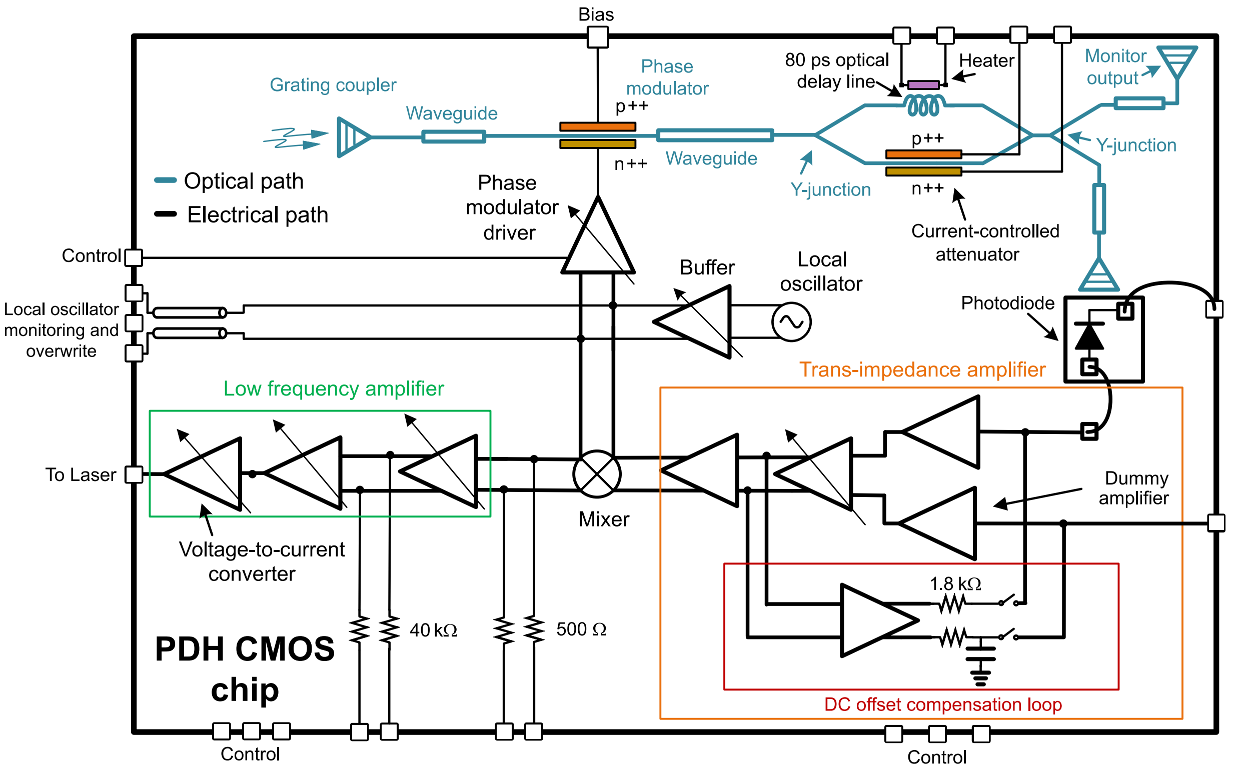

Supplementary Figure 6 | The block diagram of the Pound-Drever-Hall chip. All electronic and photonic devices and blocks are monolithically integrated on the GF7RFSOI 180 nm complementary metal-oxide semiconductor (CMOS) silicon-on-insulator (SOI) chip except for the photodiode which is hybrid integrated with the CMOS chip.

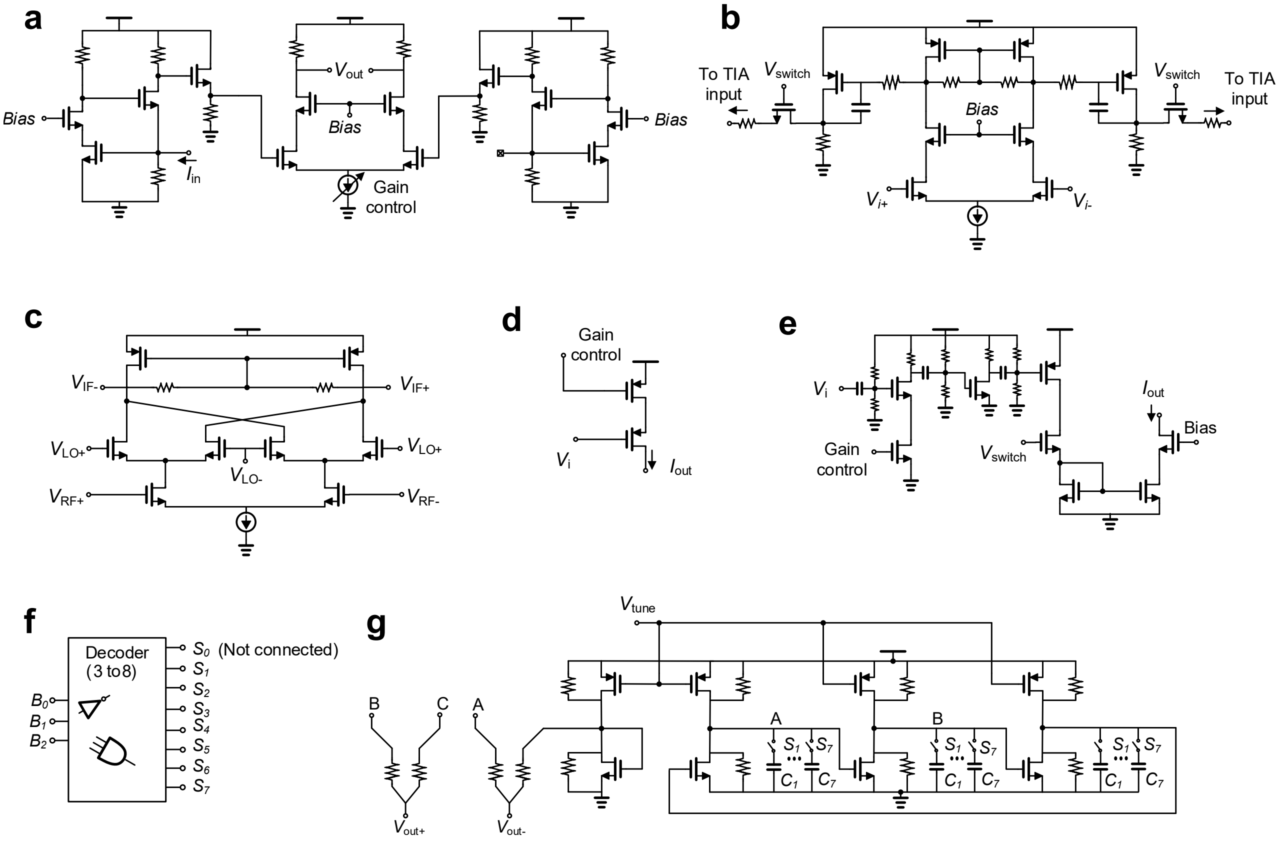

Supplementary Figure 7| The schematics of the on-chip core electronic blocks. (**a**) The trans-impedance amplifier (TIA) and the high frequency amplifier. The TIA input current, I_{in}, is amplified and converted to a differential output voltage, V_{out}. (**b**) The DC offset compensation loop. The TIA output is amplified, low-pass filtered and injected back to the TIA input to compensate for the DC offset. The feedback loop can be disabled by setting the switch voltage, V_{switch}, to zero. (**c**) Frequency mixer. The differential input radio frequency (RF) voltage, $V_{RF+} - V_{RF-}$, is mixed with the differential local oscillator (LO) voltage, $V_{LO+} - V_{LO-}$, to generate the differential intermediate frequency (IF) voltage, $V_{IF+} - V_{IF-}$. (**d**) Voltage-to-current converter (VtoI). The VtoI converts its input voltage, V_i, to a current, I_{out}, which is injected to the laser. (**e**) Phase modulator driver. The input voltage, V_i, is converted to a current, I_{out}, driving the phase modulator. The gain and phase responses of the driver can be adjusted. (**f**) Oscillator channel select circuit. A 3x8 digital decoder is used to select one of the 7 frequency channels of the oscillator, S_1 to S_7, using 3 control bits, B_0 to B_2. (**g**) The voltage-controlled ring oscillator. The channel selection for the 3-stage RF ring oscillator is performed by adjusting the switchable capacitors, $C_1$to C_7, in the oscillator stages and the fine tuning is performed by adjusting the tuning voltage, V_{tune}. The differential output of the oscillator, $V_{out+} - V_{out-}$, is buffered and routed to the modulator driver and the mixer.

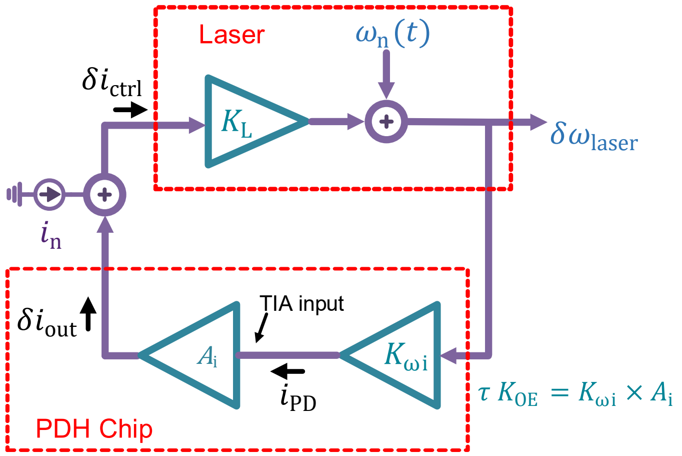

Supplementary Figure 8 | The block diagram of the linearized Pound-Drever-Hall system in presence of noise. The block diagram of the linearized Pound-Drever-Hall (PDH) system is shown where the total system noise including the noise of electronic devices and blocks, the photodiode shot noise and the relative intensity noise of the laser is modelled by a current noise, i_{n}, referred to the input of the laser. Under the closed-loop condition, the control current, δi_{ctrl}, is written as the sum of the total noise, i_{n}, and the small signal output current of the PDH chip, δi_{out}. In this case, the total frequency noise of the laser, $\delta\omega_{\mathrm{laser}}$, would be the sum of the laser intrinsic frequency noise, $\omega_{\mathrm{n}}(t)$, and the amplified current noise, $K_{\mathrm{L}} i_{\mathrm{n}}$, where K_{L} represents the small signal laser current-to-frequency conversion gain. The total laser frequency noise is amplified by the optical frequency to electrical current gain, $K_{\omega\mathrm{i}}$, and the resulting photo-current, i_{PD}, is amplified by the current gain of the electronics, A_{i}, producing the PDH chip output current. When the PDH loop is engaged, the total noise is suppressed by the loop gain, $K_{\mathrm{Loop}} = |K_{\omega\mathrm{i}} A_{\mathrm{i}} K_{\mathrm{L}}| = |\tau K_{\mathrm{OE}} K_{\mathrm{L}}|$, where τ and K_{OE} represent the delay difference between the arms of the reconfigurable Mach-Zehnder interferometer and the optical to electrical conversion gain, respectively.

Supplementary Tables

Block	Performance
Trans-impedance amplifier (TIA)	Gain: -10 to 85 dBΩ, Bandwidth: 1.55 GHz
Mixer	Conversion gain: -7 to 18 dB*, Bandwidth: 12.5 GHz*
Base-band amplifiers	Gain: 10 to 55 dB*
Oscillator	Phase noise: -116 dBc/Hz @1 MHz*, tunability: 280 MHz-3 GHz
Voltage-to-current converter	Gain:0 to 8 mA/V
Total power consumption	237 mW

*simulation

Supplementary Table 1 | The performance of the on-chip core electronic blocks. The performance of electronic blocks are summarized in this table.

Equipment	Model
RF signal generators	Agilent E4433B, Gigatronics 2520A
RF spectrum analyser	Agilent 8563E
Tunable laser	Agilent 81642A
Power supply	Keysight E3631A, Agilent 3620A
Laser current source	LDX 3620
Laser temperature controller	LDT 5412
Photodiode	Thorlabs PDB460C, MOS42AM, GT40-7002RX
Oscilloscope	Hantek DSO5102P
180° Hybrid	Minicircuits ZFSCJ-2-4-S+
Intensity modulator	UTP SN-135252A
Erbium-doped fibre amplifier (EDFA)	KPS-OEM-C-19-WDM-Sd-D-FA/02
Optical spectrum analyser	AQ6331
Wide-band laser source	Thorlabs fibre coupled SLD source
Bias tee	Picosecond Pulse labs
RF amplifier	Minicircuits ZX60-33LN-S+
Frequency counter	Tektronix FCA3120
Digital voltmeter	HP 3478A
Optical modulator driver	JDS Uniphaase H301

Supplementary Table 2 | List of equipment and components used in different experimental setups. The list of all equipment and components used for characterizations and measurements is provided.

Supplementary Notes

Supplementary Note 1: The PDH read-out signal

The block diagram of the reported Pound-Drever-Hall (PDH) system under the open-loop condition is shown in Supplementary Figure 1a where the PDH read-out signal is defined as the voltage-to-current converter (VtoI) output current, $i_{\text{out}}(t)$. Consider the case that the electric field of the laser output is written as

$$E_{\text{laser}}(t) = \sqrt{P_0}\cos(\omega_0 t), \tag{1}$$

where P_0 and ω_0 are the laser power and frequency, respectively. Also, assume the electrical oscillator output voltage can be written as

$$v_{\text{osc}}(t) = a_0 \sin(\Omega t), \tag{2}$$

where a_0 and Ω are the oscillation amplitude and frequency, respectively. The phase modulator (PM) driver increases the level of the oscillator output to v_0 and introduces a phase shift, θ, between its input and output. In this case, the modulator driver output is written as

$$v_{\text{PM,driver}}(t) = v_0 \sin(\Omega t + \theta). \tag{3}$$

The modulation index can be defined as

$$\beta = \frac{\left(\frac{v_0}{Z_{\text{in,mod}}}\right)}{i_\pi}\pi, \tag{4}$$

where i_π is the current required to generate a π radians optical phase shift across the optical phase modulator and $Z_{\text{in,mod}}$ represents the input impedance of the phase modulator. In this case, the electric field at the output of the phase modulator is written as

$$E_{\text{mod}}(t) = \sqrt{P_0}\cos[\omega_0 t + \beta\sin(\Omega t + \theta)]. \tag{5}$$

The modulated signal passes through the electronically reconfigurable Mach-Zehnder interferometer (MZI). The MZI output is photo-detected and the photo-current is written as

$$i_{\text{PD}}(t) = \frac{1}{4}RP_0\alpha\{\cos[\omega_0(t-\tau) + \beta\sin(\Omega(t-\tau)+\theta)] + \cos[\omega_0 t + \phi + \beta\sin(\Omega t + \theta)]\}^2, \tag{6}$$

where R, α, τ and ϕ are the photodiode responsivity, the total optical transmission, the delay difference between the two arms of the MZI and the relative phase between the two arms of the MZI, respectively. The photo-current can be simplified to

$$i_{\text{PD}}(t) = \frac{1}{4}RP_0\alpha\{\cos(\omega_0\tau + \phi)\cos[Z\cos(\Omega t + \psi)] - \sin(\omega_0\tau + \phi)\sin[Z\cos(\Omega t + \psi)]\} + DC\ terms, \quad (7)$$

where

$$Z = 2\beta \sin\left(\frac{\Omega\tau}{2}\right) \text{ and } \psi = \theta - \frac{\Omega\tau}{2}. \quad (8)$$

Using the Jacobi-Anger expansion[1], the photo-current is written as

$$i_{\text{PD}}(t) = \frac{1}{2}RP_0\alpha \sin(\omega_0\tau + \phi) \sum_{n=1}^{\infty}\{(-1)^n J_{2\text{n}-1}(Z)\cos[(2n-1)(\Omega t + \psi)]\} + \text{DC terms} + \text{terms at } 2n\Omega, \quad (9)$$

where $J_{2\text{n}-1}(.)$ represent the Bessel functions of the first kind. The photo-current is converted to a voltage and amplified using a trans-impedance amplifier (TIA) and mixed with $v_{\text{osc}}(t)$. The mixer output is low-pass filtered (to remove higher order terms), amplified and converted to a current using a VtoI. In this case, the output current of the VtoI, the read-out signal, can be written as

$$i_{\text{out}}(t) = K_{\text{OE}} \times \sin(\omega_0\tau + \phi), \quad (10)$$

where K_{OE} is calculated as

$$K_{\text{OE}} = \frac{1}{2}RP_0\alpha J_1(\beta\Omega\tau)\sin\left(\theta - \frac{\Omega\tau}{2}\right)A_{\text{i}}. \quad (11)$$

In Supplementary Equation (11), $A_{\text{i}} = \frac{i_{\text{out}}}{i_{\text{PD}}} = G_{\text{TIA}}G_{\text{Mixer}}G_{\text{VtoI}}$ represents the total electrical current gain which is defined as the ratio of the VtoI output current to the photo-current. Also, G_{TIA}, G_{Mixer} and G_{VtoI} are the TIA trans-impedance gain, the mixer conversion gain and the VtoI trans-conductance gain, respectively. Note that since the on-chip delay τ is much smaller than Ω^{-1}, in the derivation of Supplementary Equation (11), it is assumed that $2\beta\sin\left(\frac{\Omega\tau}{2}\right) \approx \beta\,\Omega\tau$.

Supplementary Figure 1b shows the simulated read-out signal and the simulated MZI transfer function. As shown, the read-out signal is asymmetric with respect to the frequency of the notch in the MZI frequency response, f_{notch}. Therefore, the read-out signal indicates both the difference between the laser frequency and f_{notch} and whether the laser frequency is greater or less than f_{notch}. Thus, by injecting the read-out signal to the laser, the laser frequency can be locked to the frequency of the notch in the MZI.

Supplementary Note 2: Characterization of the integrated phase modulator

Supplementary Figure 2 shows the cross-section of the implemented p-doped-intrinsic-n-doped (PIN) phase modulator which is similar to a previously reported PIN design[2]. The electrical characterization shows that at 2.0 V bias voltage, the input impedance of the PIN can be modelled as an 8 Ω resistor in parallel with a 133 pF capacitor. Supplementary Figure 3a shows the measurement setup used for insertion loss characterization of the PIN structure as a function of the bias current. An optical probe is used to couple the output of the Agilent 81642A tunable laser into the PIN test structure using the on-chip grating coupler. The test structure output is coupled into an optical fibre and used to measure the optical power. A current source is used to sweep the control current of the PIN structure. The measured insertion loss of the PIN modulator versus current (at 1540 nm) is shown in Fig. 2i. Supplementary Figure 3b shows the measurement setup used for bandwidth characterization. The same reconfigurable MZI used in the PDH system is used for phase modulator characterization. First, the wavelength of the tunable laser and the RF signal power were set to 1540 nm and 0 dBm, respectively. Then, the frequency of the Agilent E4433B RF synthesizer was swept and the output of the test structure was photo-detected, amplified and recorded using an Agilent 8563E spectrum analyser where a 3-dB bandwidth of 150 MHz was measured (Fig. 2g).

The setup shown in Supplementary Figure 3b can also be used for i_π characterization. Consider the case that the laser electric field and the RF signal are written as $E_{\mathrm{i}} = \sqrt{P_0}e^{j\omega_0 t}$ and $v_{\mathrm{mod}} = v_0 \sin(\Omega t)$, where P_0, ω_0, v_0 and Ω are the laser power, the laser frequency, the RF amplitude and the RF frequency, respectively. Assuming that the relative phase between the arms of the MZI is set to 90°, the electric field at the output of the MZI can be written as

$$E_{\mathrm{out}} = \frac{\sqrt{P_0}}{2}\, e^{j\omega_0 t}\left\{j + \sqrt{\alpha}e^{j\beta \sin(\Omega t)}\right\}, \tag{12}$$

where α and β represent the optical transmission (in one arm of the MZI) and the modulation index ($\beta \triangleq \frac{v_0}{i_\pi |Z_{\mathrm{in}}|}\pi$), respectively. Note that Z_{in} represents the input impedance of the PIN structure. The photo-current in Supplementary Figure 3b is written as

$$i_{\mathrm{PD}}(t) = R|E_{\mathrm{out}}|^2 = \frac{P_0 R}{4}\left\{1 + \alpha + 2\sqrt{\alpha}\sin(\beta \sin(\Omega t))\right\}, \tag{13}$$

where R represents the responsivity of the off-chip photodiode in the measurement setup. Using the Jacobi-Anger expansion[1] , the AC component of the photo-current is written as

$$i_{\text{PD,ac}}(t) = P_o R \sqrt{\alpha} \sum_{n=1}^{\infty} J_{2n-1}(\beta) \sin\big((2n-1)\Omega t\big). \tag{14}$$

Therefore, at a given frequency, by fitting a Bessel function of the first kind to the measured AC component of the photo-current, the modulation index and hence i_π can be estimated. Supplementary Figure 3c shows the modulator photo-current (amplified and monitored on an electrical spectrum analyzer) measured at 150 MHz for different modulator currents where an i_π of about 64 mA is estimated. We have repeated this measurement at different frequencies to plot Fig. 2h.

Supplementary Note 3: Characterization of the integrated electronically reconfigurable Mach-Zehnder interferometer

The measurement setup for the integrated reconfigurable MZI is depicted in Supplementary Figure 4. The output of an Agilent 81642A tunable laser is amplified using an Erbium-doped fibre amplifier (EDFA) and coupled into the reconfigurable MZI test structure using an on-chip grating coupler. The output of the MZI test structure is photo-detected, amplified and monitored using a HP 3478A digital voltmeter. By adjusting the current injected to the PIN current-controlled attenuator, the loss mismatch between the two arms of the MZI can be minimized resulting in more than 28 dB extinction ratio as shown in Fig. 3b. Once the loss mismatch between the two arms of the MZI is minimized, the relative phase between the two arms can be adjusted using the heaters placed between the waveguides of the delay line.

Supplementary Note 4: The frequency noise discriminator

Supplementary Figure 5a shows the schematic of the optical fibre based MZI with 8.5 cm length imbalance used as a frequency discriminator in Fig. 6a. A thermoelectric cooler (TEC) is placed under the 8.5 cm delay line to set the relative phase between the two arms of the MZI to 90°. The MZI output is split into two branches using a 50/50 fusion coupler. The bottom branch is photo-detected, amplified and used to monitor the frequency noise of the laser using an Agilent 8563E spectrum analyser. The top branch is used to adjust the relative phase between the two arms of the fibre based MZI using a control unit. The output of the control unit is monitored on an oscilloscope to ensure accurate relative phase adjustment (Fig. 6a).

Supplementary Figure 5b shows the schematic of the control unit used to set the relative phase between the two arms of the off-chip optical fibre based MZI (the frequency discriminator). Supplementary Figure 5c shows the output

voltage of the control unit which corresponds to the relative phase between the two arms of the frequency discriminator. When the phase control loop is closed, the phase difference between the two arms can be set to 90°.

Supplementary Note 5: The CMOS chip details

The block diagram of the reported PDH chip is shown in Supplementary Figure 6 where all photonic and electronic devices and blocks are monolithically integrated on a CMOS chip except for the photodiode which is hybrid integrated (vertically) with the CMOS chip. The laser output is coupled into the chip using a grating coupler and phase modulated using a PIN phase modulator. The modulated optical signal is guided to the electronically reconfigurable MZI. Control pads are used to adjust the loss in the bottom arm of the MZI using the PIN current-controlled attenuator and the relative phase between the two arms using heaters placed between the delay lines in the top arm. The MZI output is photo-detected using the photodiode chip placed on top of the grating coupler. The photo-current is wire-bonded back to the CMOS chip and amplified and converted to a voltage using a multi-stage TIA. The gain of the TIA can be adjusted using control pads. An offset cancellation loop is designed to compensate for the offset voltage generated as a result of process variation and thermal fluctuations. The TIA output is mixed with the LO signal using a double-balanced mixer. The gain of the mixer can also be controlled using control pads. The mixer output is amplified and low-pass filtered using a multi-stage amplifier with adjustable gain and bandwidth. A VtoI converts the amplifier output voltage to a current which is injected to the laser gain section. The gain of the VtoI can be adjusted using control pads. The output of the on-chip oscillator, the LO signal, is buffered and used to drive the phase modulator (using the modulator driver) and the LO port of the mixer. Signal-ground-signal (SGS) pads are used to monitor the on-chip oscillator output. These pads can also be used to overwrite the on-chip oscillator. The gain and phase responses of the on-chip phase modulator driver can be adjusted through control pads. The schematics of core electronic blocks of the CMOS chip are depicted in Supplementary Figure 7. The entire PDH system consumes 237 mW.

Supplementary Note 6: Characterization of the CMOS electronic blocks

All electronic blocks have been integrated on the GlobalFoundries GF7RFSOI process, a standard CMOS SOI technology, with no post-processing. The performance of the core electronic blocks (shown in Supplementary Figure 7) is summarized in Table 1.

Supplementary Note 7: The noise sources

The key noise sources affecting the PDH stabilization performance are the relative intensity noise (RIN) of the laser, the photodiode shot noise and the noise of the electronic devices. The effect of each of these noise sources can be modelled as a current noise referred to the input of the TIA[3]. Since these noise sources are independent, the total noise contribution can be written as the sum of these three current noises.

In presence of laser intensity noise, Supplementary Equation (1) describing the electric field of the laser output, can be modified to

$$E_{\text{laser}}(t) = \sqrt{P_0 + p_{\text{n}}(t)}\cos(\omega_0 t), \tag{15}$$

where P_0, $p_{\text{n}}(t)$ and ω_0, are the laser power, laser intensity noise and the lasing frequency, respectively. In this case and following the same steps taken in derivation of Supplementary Equation (7), the photodiode current can be calculated as

$$\begin{aligned} i_{\text{PD}}(t) \approx\ & i_{\text{DC}} + \frac{\alpha R}{8}\big(p_{\text{n}}(t) + p_{\text{n}}(t-\tau)\big) \\ & + \frac{\alpha P_0 R}{4}\left(1 + \frac{\big(p_{\text{n}}(t) + p_{\text{n}}(t-\tau)\big)}{2P_0}\right)\cos\big(\omega_0\tau + Z\cos(\Omega t + \psi)\big) \end{aligned} \tag{16}$$

where $i_{\text{DC}}, \alpha, R, \tau$ and Ω are the DC component of the photo-current, the transmission of the optical path, the responsivity of the photodiode, the delay difference between the two arms of the reconfigurable MZI and the modulation frequency, respectively. Also, Z and ψ are defined in Supplementary Equation (8). Note that due to the amplitude limiting mechanism, the amplitude noise of the local oscillator is negligible[4] and is ignored in this analysis. Assuming $\frac{(p_{\text{n}}(t)+p_{\text{n}}(t-\tau))}{2P_0} \ll 1$, Supplementary Equation (16) can be written as

$$i_{\text{PD}}(t) \approx i_{\text{DC}} + \frac{\alpha R}{8}\big(p_{\text{n}}(t) + p_{\text{n}}(t-\tau)\big) + \frac{\alpha P_0 R}{4}\cos\big(\omega_0\tau + Z\cos(\Omega t + \psi)\big). \tag{17}$$

Assuming $p_{\text{n}}(t)$ to be an additive mean-zero white Gaussian process, the power spectral density (PSD) of the laser intensity noise equivalent current, i_{RIN}, can be written as[3]

$$\overline{i_{\text{RIN}}^2} = R^2\overline{[p_{\text{n}}(t) + p_{\text{n}}(t-\tau)]^2} = 2R^2P_0^2\text{RIN}, \tag{18}$$

where $\text{RIN} = \frac{\overline{p_{\text{n}}^2}}{P_0^2}$ represents the relative intensity noise of the laser. Therefore, using Supplementary Equation (18) and considering the shot noise of the photodiode, $i_{\text{PD,shot}}$, Supplementary Equation (17) is modified to

$$i_{\text{PD}}(t) \approx i_{\text{DC}} + i_{\text{PD,shot}} + \frac{\alpha}{8}i_{\text{RIN}} + \frac{\alpha P_0 R}{4}\cos\big(\omega_0\tau + Z\cos(\Omega t + \psi)\big). \tag{19}$$

The noise contribution of electronic devices can be modelled as a current noise referred to the output of the photodiode[3]. In this case, the total photo-current including the effect of all aforementioned noise sources is written as

$$i_{\mathrm{PD}}(t) \approx i_{\mathrm{DC}} + i_{\mathrm{noise,total}} + \frac{\alpha P_0 R}{4}\cos\big(\omega_0 \tau + Z\cos(\Omega t + \psi)\big), \tag{20}$$

where $i_{\mathrm{noise,total}} = i_{\mathrm{PD,shot}} + \frac{\alpha}{8} i_{\mathrm{RIN}} + i_{\mathrm{n,electronics}}$, represents the total input referred current noise and $i_{\mathrm{n,electronics}}$ represents the noise of the electronic devices. Since the total current noise is a small signal, the linearized PDH system in Supplementary Figure 8 can be used for noise analysis. Note that in this figure, the PDH chip gain (*i.e.* τK_{OE} in Fig. 5) is written as the product of an optical frequency to current gain, $K_{\omega\mathrm{i}}$, and the electrical current gain, A_{i} (introduced in Supplementary Note 1). Using Supplementary Equation (11), the optical frequency to current gain is defined as $K_{\omega\mathrm{i}} \triangleq \frac{1}{2}\tau R P_0 \alpha J_1(\beta\Omega\tau)\sin\left(\theta - \frac{\Omega\tau}{2}\right)$. In this case, the total current noise referred to the output of the CMOS chip, i_{n} in Supplementary Figure 8, is written as

$$i_{\mathrm{n}} = A_{\mathrm{i}} \times\, i_{\mathrm{noise,total}} = A_{\mathrm{i}} \times \left(i_{\mathrm{PD\,,shot}} + \frac{\alpha}{8} i_{\mathrm{RIN}} + i_{\mathrm{n,electronics}}\right). \tag{21}$$

The control current injected to the laser in Supplementary Figure 8 is written as

$$\delta i_{\mathrm{ctrl}} = \delta i_{\mathrm{out}} + i_{\mathrm{n}}. \tag{22}$$

In this case, when the PDH loop is closed, the total laser frequency noise is written as

$$\delta\omega_{\mathrm{laser}} = \frac{\omega_{\mathrm{n}}(t)}{1+K_{\mathrm{Loop}}} + \frac{K_{\mathrm{L}}}{1+K_{\mathrm{Loop}}} i_{\mathrm{n}}, \tag{23}$$

where $K_{\mathrm{Loop}} = |K_{\omega\mathrm{i}} A_{\mathrm{i}} K_{\mathrm{L}}| = |\tau K_{\mathrm{OE}} K_{\mathrm{L}}|$ is the PDH loop gain and $\omega_{\mathrm{n}}(t)$ represents the intrinsic frequency noise of the laser. Supplementary Equation (23) indicates that when the PDH stabilization loop is engaged, the contribution of the electro-optic noise (the noise of electronic devices, the photodiode shot noise and the relative intensity noise of the laser) to the laser frequency noise is suppressed by the loop gain.

Supplementary Note 8: Hybrid integration of the photodiode and the CMOS chip

Despite successful monolithic integration of all active and passive photonic and electronic devices on the GF7RFSOI process, a wideband photodiode with high responsivity was not implemented as no material with efficient absorption coefficient in the 1550 nm range is available in this standard electronic process. Therefore, a photodiode chip is hybrid integrated (vertically) with the CMOS chip as depicted in Figs. 1b and 1c.

A Cosemi LX3050 InGaAs/InP PIN photodiode chip with a die area of 450 μm x 450 μm and thickness of 152 μm was mounted on top of the CMOS chip. The active area of this vertically illuminated photodiode is about 800 μm^2 (diameter of about 32 μm) which is more than 4 times larger than the grating coupler area (in Fig. 2a) resulting in a relatively relaxed alignment tolerance. To hybrid integrate the photodiode with the CMOS chip, the photodiode was placed on top of the CMOS chip and was moved using a DC probe and aligned with the grating coupler using markers. Once the photodiode was aligned with the grating coupler, a drop of Loctite Instant Mix epoxy was placed at the corner of the CMOS chip flowing and surrounding the photodiode chip. When the epoxy was cured, the photodiode pads were wire-bonded to the input pads of the CMOS chip as shown in Fig. 1c. Note that the photodiode can be backside-illuminated since its substrate is substantially transparent at 1550 nm. This process that we have developed previously[5] is reliable and repeatable. The measured average coupling loss is under 1.8 dB which is lower than the coupling loss between a grating coupler and an optical fibre in the GF7RFSOI process (Fig. 2b).

Supplementary References

1. Colton, D. & Kress, R. Inverse Acoustic and Electromagnetic Scattering Theory Ch. 2. (Springer Science & Business Media, New York, 2012).

2. Khachaturian, A., Abiri, B., Zhou, A. & Hajimiri, A. Monolithic Mach-Zehnder Interferometer Modulator in an unmodified CMOS process. *IEEE Photonics Conference (IPC)*, 394-395 (2015).

3. Aflatouni, F., Bagheri, M. & Hashemi, H. Design methodology and architectures to reduce the semiconductor laser phase noise using electrical feedforward schemes. *IEEE Trans. Microw. Theory Techn.* **58**, 3290-3303 (2010).

4. Hajimiri, A. & Lee, T. H. A general theory of phase noise in electrical oscillators. *IEEE J. Solid-State Circuits* **33**, 179-194 (1998).

5. Afiatouni, F*., et al.* Electronic laser phase noise reduction. *IEEE RFIC Symp.*, 265-268 (2013).
